# Effect of lifestyle or metformin interventions before IVF/ICSI treatment on infertile women with overweight/obese and insulin resistance: a factorial design randomised controlled pilot trial

**DOI:** 10.1186/s40814-023-01388-x

**Published:** 2023-09-12

**Authors:** Xiaojuan Wang, Sufen Cai, Sha Tang, Lanlin Yang, Jing Tan, Xin Sun, Fei Gong

**Affiliations:** 1 Department of Epidemiology and Health Statistics, School of Public Health, Central South University, Changsha, 410008 Hunan China; 2 Clinical Research Center for Reproduction and Genetics in Hunan Province, Reproductive and Genetic Hospital of CITIC-XIANGYA, Changsha, 410008 Hunan China; 3 Chinese Evidence-Based Medicine Center, West China Hospital, Sichuan University, Chengdu, 610041 Sichuan China; 4 NMPA Key Laboratory for Real World Data Research and Evaluation in Hainan, Chengdu, 610041 Sichuan China; 5 Laboratory of Reproductive and Stem Cell Engineering, Key Laboratory of National Health and Family Planning Commission, Central South University, Changsha, 410008 Hunan China

**Keywords:** In vitro fertilisation, Obesity, Insulin, Life style, Metformin, Factorial design

## Abstract

**Background:**

For infertile women with overweight/obesity and insulin resistance (IR), it is uncertain whether intervention before infertility treatment can improve live birth rate (LBR). We implemented a factorial-design study to explore the effectiveness of lifestyle and metformin interventions. This pilot study aimed to evaluate the feasibility of a definitive study.

**Methods:**

We randomised 80 women without polycystic ovarian syndrome (PCOS) who planned to start their first or second IVF/ICSI treatment with a body mass index ≥ 25 kg/m^2^ and IR. Participants were randomised (1:1:1:1) into four groups: (A) lifestyle intervention, (B) metformin intervention, (C) lifestyle + metformin intervention, or (D) no intervention. All interventions were performed before IVF/ICSI treatment.

**Results:**

During 10 months, 114 women were screened and eligible; 80 were randomised, and 72 received the assigned treatment. The recruitment rate was 70.18% (80/114, 95% CI 61.65%–78.70%). An average of 10 participants were randomised each month. None of the participants crossed over from one group to another. Approximately 93.15% (68/73) of the participants achieved good intervention compliance. Only 77.78% (56/72) of the recruited participants started infertility treatment after achieving the goal of the intervention. All randomised participants completed the follow-up. Mild adverse events after metformin administration were reported in 43.24% (16/37) of the cases, although no serious adverse events related to the interventions occurred. The LBR for groups A + C and B + D were 33.33% (12/36) and 33.33% (12/36) (RR = 1.00, 95%CI:0.52–1.92) (lifestyle intervention effect). The LBR for groups B + C and A + D were 43.24% (16/37) and 22.86% (8/35) (RR = 1.89, 95% CI:0.93–3.86) (metformin intervention effect). There was no evidence for an intervention interaction between lifestyle and metformin. We cannot yet confirm the effects of lifestyle, metformin, or their interaction owing to the insufficient sample size in this pilot study.

**Conclusions:**

Instituting a 2 × 2 factorial design randomized controlled trial (RCT) is feasible, as the pilot study showed a high recruitment rate and compliance. There is no evidence that lifestyle or metformin improves live birth, and adequately powered clinical trials are required.

**Trial registration:**

clinicaltrials.gov NCT03898037. Registered: April 1, 2019.

**Supplementary Information:**

The online version contains supplementary material available at 10.1186/s40814-023-01388-x.

## Key messages regarding feasibility

- What uncertainties existed regarding the feasibility?
  For infertile women with overweight/obesity and insulin resistance, it is uncertain whether lifestyle or metformin intervention before infertility treatment can improve live birth rate. We intended to implement a factorial design to explore the effectiveness of lifestyle or metformin interventions and the interaction effect of the two interventions. First, this target population has been poorly studied, and we are not sure whether a sufficient number of subjects can be recruited. Second, receiving lifestyle or metformin intervention means that subjects need to exercise and diet or take metformin according to the study protocol, and participants would start IVF/ICSI treatment only after achieving the goals of the interventions. We are unsure whether the participants will comply with the intervention. Third, the primary clinical outcome was live birth, with post-pregnancy outcomes such as live birth, miscarriage, and pregnancy complications were followed-up by telephone, and follow-up compliance was uncertain.
- What are the key feasibility findings?
  The pilot study enrolled sufficient participants within the planned time, and both intervention and follow-up compliance could be achieved as expected.
- What are the implications of the feasibility findings of the main study?
  The pilot study found that the eligibility rate was low, suggesting that researchers could consider relaxing the conditions for enrolment in main study, such as removing some exclusion criteria. In addition, stratified randomization should be used in order to better balance basic characteristics between groups.

## Background

The prevalence of infertility among women 20–44 years is about 12.5% worldwide [1]. Approximately 30% of women with infertility are reported to be overweight or obese [2]. Overweight or obese women undergoing in-vitro fertilization/ intracytoplasmic sperm injection (IVF/ICSI) treatment require higher doses of gonadotropins, illustrating an impaired response to ovarian stimulation as well as poor oocyte and embryo quality [3], thus increasing miscarriage rates [4, 5]. Previous studies have indicated that overweight and obese women can release adipokines (such as leptin and adiponectin) and pro-inflammatory cytokines (such as TNF-α, IL-1, and IL-6), which could directly affect the hypothalamic-pituitary–gonadal (HPG) axis or the endometrium, oocyte, or embryo itself, thus further reducing the success rate of IVF/ICSI treatment [5–7]. On the other hand, adipokines and proinflammatory cytokines can cause metabolic abnormalities, such as insulin resistance (IR) [8]. IR is a common disorder among overweight and obese populations and is considered an important prognostic factor for IVF/ICSI treatment [9, 10]. In theory, overweight/obesity with IR has a greater negative effect on IVF/ICSI outcomes than overweight/obesity without IR. We intend to select infertile women with overweight/obesity and IR as our research subjects and take interventions against overweight/obesity and IR to explore whether these interventions can improve pregnancy outcomes.

To improve the success rate of IVF/ICSI in infertile patients who are overweight or obese, multiple intervention approaches have been adopted in clinical practice, including lifestyle interventions, medication, and weight loss surgery. Lifestyle intervention, as the first-line approach in overweight or obesity management guidelines [11, 12], has been shown to reduce weight, improve insulin levels, normalise menstruation, improve ovulation, and increase the spontaneous pregnancy rate [13, 14]. Although current evidence supports the idea that lifestyle interventions may improve ovarian function, whether lifestyle interventions can improve the live birth rate(LBR) in infertility treatment remains inconclusive [15, 16]. Receiving lifestyle interventions implies delaying infertility treatment. There is still a tradeoff between the benefits of lifestyle interventions and the impact of delayed infertility treatments in infertile women.

Metformin, an insulin sensitisation agent, has also been reported to have a modest effect on weight loss [17] and is used in women with polycystic ovary syndrome (PCOS). Some studies have shown that the use of metformin combined with ovulation stimulants can improve ovulation and clinical pregnancy rates in patients [18, 19]. However, there is no high-quality evidence to support that metformin improves the LBR in patients with PCOS. Owing to the complex pathogenic mechanisms and diverse phenotypes of PCOS, previous studies on PCOS are heterogeneous. In non-PCOS patients, it may be easier to explore the efficacy of metformin on IR in infertile patients, as well as its effect on live births after infertility treatment. Few studies have investigated metformin in non-PCOS patients with overweight/obesity or IR. Whether metformin intervention improves pregnancy outcomes in patients without PCOS with obesity or IR remains unclear. In addition, both lifestyle and metformin interventions could reduce weight and regulate insulin levels; however, research on the effect of combined interventions and the interaction of the two interventions is rare.

To evaluate whether lifestyle and/or metformin interventions before infertility treatment can improve LBR in non-PCOS patients with infertility and IR, we plan to conduct a 2 × 2 factorial randomised controlled trial (RCT). As we were uncertain whether enough participants could be recruited and whether the participants would comply with the interventions and follow-up, we conducted this pilot study to assess whether a formal trial was feasible.

## Methods

### Study design

We conducted a 2 × 2 factorial design, randomised, unblinded, external pilot trial at the Reproductive and Genetic Hospital of CITIC-Xiangya from June 2019 to June 2021. All the participants provided written informed consent. The trial was approved by the hospital ethics committee (Approved ID: LL-SC-2019–001) and registered at ClinicalTrials. gov of the United States National Institutes of Health (NCT03898037). The study was reported in accordance with the extended guidelines of the Consolidated Standards of Reporting Trials (CONSORT) extended guideline for pilot and feasibility trials.

### Study population

Eligible participants were non-PCOS infertile women between 18 and 36 years of age with indications for IVF/ICSI treatment, planning to start their first or second IVF/ICSI treatment, with a BMI ≥ 25 kg/m^2^ and IR. IR was assessed using the homeostasis model assessment (HOMA), and HOMA-IR was calculated as fasting glucose (FPG, mmol/L) multiplied by fasting insulin (FINS, mIU/mL) divided by 22.5. A HOMA value greater than 2.69 was used to indicate IR. PCOS was diagnosed using the modified Rotterdam criteria [20, 21], which include menstrual abnormalities (irregular uterine bleeding, oligomenorrhea, or amenorrhoea) combined with either hyperandrogenism or polycystic ovaries, as validated in the Chinese population [22].

Patients were excluded if they had any of the following conditions: endometriosis, congenital or acquired uterine abnormalities (e.g. uterine malformation, adenomyosis, submucosal myoma, or intrauterine adhesion), planned oocyte donation, planned preimplantation genetic tests (PGT), endocrinologic or metabolic diseases (e.g. diabetes mellitus, Cushing syndrome, congenital adrenal hyperplasia, pituitary amenorrhoea, or thyroid dysfunction), a history of recurrent spontaneous abortion (defined as three or more previous spontaneous pregnancy losses), bad compliance to follow verbal and written instructions, or specific dietary/drug intervention for the past 3 months.

### Randomisation

All patients were prescreened by research nurses. Once eligibility was confirmed, clinicians and research nurses conducted face-to-face interviews with the participants to obtain written informed consent and baseline data. An independent statistician used the block randomisation method (group numbers were 4, the distribution ratio was 1:1:1:1, and the block size was set at 8) to generate a randomised sequence by SAS V9.2 (SAS. Cary, NC, USA). The statistician then sealed the random order and group names into opaque envelopes. The randomisation sequence was maintained by the statisticians. The authorized investigator assigned participants to different groups by sequentially opening the envelopes.

### Interventions

The goal of the lifestyle intervention was to lose 5–10% of body weight during the intervention period, and the goal of the metformin intervention was to improve IR and reduce HOMA values to below 2.69. The combined intervention group (lifestyle and metformin interventions) met both criteria. The maximum intervention duration was 12 weeks. To enhance adherence to the intervention, once the women achieved their goal, they could start their IVF/ICSI treatment even before the end of the 12 weeks. All participants started IVF/ICSI treatment after the 12-week intervention, regardless of the goal achieved.

Height, weight, waist-hip circumference, OGTT, and IRT were measured on the day of randomisation (baseline) and at 4, 8, and 12 weeks after randomisation in the three intervention groups, whereas the above indices were measured only on the day of randomisation and before IVF/ICSI treatment in the no intervention group.

The participants assigned to the lifestyle intervention group were guided by trained dietitians prior to the trial. Women were advised to reduce their energy intake by 500 kcal with the help of a diary, while maintaining a minimum caloric intake of 1200 kcal per day. They were also advised to engage in moderate-intensity physical activity with a target level of 10,000 steps per day and at least 30 min of moderate-intensity exercise two or three times per week. Dietitians encouraged participants to use the “Weight Steward” application (APP) [23] to record daily diet, exercise, and weight in different ways such as text, voice, picture, and video for self-monitoring, at the same time, dietitians conducted regular follow-up and provided guidance through the APP.

Participants allocated to the metformin intervention group were administered metformin (metformin hydrochloride tablets, 500 mg; Sino American Shanghai Squibb Pharmaceuticals Ltd, Shanghai, China) at an initial dose of 500 mg bid for the first week and then increased thereafter to 500 mg tid for 12 weeks. When participants reported discomfort such as nausea, vomiting, anorexia, or diarrhoea, the drug dosage was reduced until they could tolerate it.

In the combination intervention group, lifestyle and metformin were administered in a similar way. The non-intervention group did not require lifestyle or metformin intervention prior to IVF treatment.

### IVF/ICSI treatment

All patients were treated using the long luteal GnRH agonist protocol described by Tan et al. [24]. A 1.5 mg dose of the GnRH analogue triptorelin (Decapeptyl; Ferring, Malmo, Sweden) was administered in the mid-luteal phase of the menstrual cycle. After complete desensitisation, 112.5–375.0 IU of recombinant Follicle-Stimulating Hormone (FSH) (Gonal-F, Merck-Serono, Geneva, Switzerland; Puregon, NV Organon, Oss, The Netherlands) and/or human menopausal gonadotropins (hMG, Lizhu, China) were administered daily until the day of human chorionic gonadotropin (HCG) administration. The starting dose of gonadotropins was based on the patient’s age, body weight, ovarian reserve test results, and/or previous response to ovarian stimulation. HCG (5000–10000 IU, Pregnyl; Merck) was injected when at least two follicles reached 18 mm in size. Oocyte retrieval was performed 34–36 h later under general anaesthesia using intravenous propofol (Astra Zeneca, UK Ltd.). Eggs were fertilised by IVF or, in the case of ICSI, 4–6 h after oocyte retrieval, and normal fertilisation was identified 16–18 h after injection by the presence of two pronuclei and two polar bodies.

On day 3, the embryos were scored using the Puissant criteria and transferred. Luteal phase support was provided from the day of oocyte retrieval using Crinonew progesterone gel (Columbia Laboratories, Inc., Livingston, NJ, USA) until pregnancy test which was performed 14 days after embryo transfer (ET). If the patient was pregnant, defined as a serum HCG level > 10 IU/L, vaginal sonography was performed 4 weeks after ET to confirm clinical pregnancy, and ongoing pregnancy was confirmed by transvaginal ultrasound at approximately 10 weeks of gestation. At 28 weeks gestation, 37 weeks gestation and 4 weeks after delivery, pregnancy complications and neonatal outcomes were followed up through telephone call and relevant medical records were requested to be transmitted to investigators online.

### Study outcomes

#### Feasibility outcomes

The primary outcome of feasibility was the recruitment rate (The number of participants randomized/the number of participants assessed for eligibility). We expect a recruitment rate of at least 60%. By referring to methodological issues for feasibility and the pilot study recommended by Shanyinde et al. [25] and considering the progress and quality of the study, we set several feasibility criteria as follows:

1. At least 10 subjects were randomised each month on average;
2. No more than 5% of the recruited participants crossed over from one group to another;
3. At least 80% of all recruited subjects presented good compliance (good compliance with metformin intervention means that the actual metformin dose accounts for 80–120% of the recommended dose; good compliance with lifestyle interventions was evaluated by a dietitian based on daily energy intake and exercise) to implement interventions;
4. At least 80% of all recruited participants started IVF/ICSI infertility treatment after the goal of the intervention was achieved or after a 12-week intervention was completed.
5. At least 95% of the recruited participants completed their final follow-up.

#### Clinical outcomes

The primary clinical outcome of the RCT was live birth, defined as the delivery of any viable infant at 28 weeks or more of gestation.

The pre-specified secondary outcomes were pregnancy-related measurements such as spontaneous pregnancy, clinical pregnancy, and miscarriage. We also investigated intervention-related measurements, including weight loss percentage (weight loss from baseline to end of intervention/baseline weight), weight loss of 5–10%, reduced waist circumference, reduced hip circumference, and homeostatic model assessment (HOMA) value below 2.69, as well as IVF-related measurements, including number of cancelled cycles, total dose of gonadotropins, number of oocytes retrieved, and number of embryos.

Adverse outcomes in women included complications due to infertility treatment (such as ovarian hyperstimulation syndrome and ectopic pregnancy) and adverse events related to drugs or weight loss (including nausea, vomiting, diarrhoea, and constipation).

### Statistical considerations

#### Sample size

Using a conservative estimate of 40% of LBR with lifestyle or metformin intervention based on previous trials and assuming that a 15% absolute increase in LBR would be clinically important, we calculated that at least 342 patients (171 in each of the two treatments) would provide 80% power to detect this difference (a = 5%).

In this pilot study, we used a confidence interval (CI) approach to estimate the sample size of pilot study [26]. The expected recruitment rate (primary outcome of feasibility) was 60%. We set a margin of error (ME) of 0.05, a lower limit of the CI of 0.60, and an expected recruitment rate of 70%, based on the formula $\mathrm{p}\pm \mathrm{z\alpha }\sqrt{p(1-p)/n}$, the required sample for the pilot study should be at least 78 patients. To ensure that the four groups were evenly distributed, we randomised the group allocations of the 80 women. It is worth noting that this is an appropriate sample size for feasibility assessment [27] but not sufficiently powered to detect differences in clinical outcomes.

#### Statistical analysis

Continuous data were described as means and standard deviations (SD) for normally distributed data and as medians and interquartile ranges for skewed data. Categorical data were reported as absolute numbers and percentages. Differences were tested using the Mann–Whitney U test for continuous variables and the chi-square test for categorical variables. The relative risk (RR) for each intervention was calculated by dividing the incidence of outcomes in the intervention group by the incidence of outcomes in the non-intervention group, and Risk Estimate of Crosstabs (SPSS process) was used to calculate the 95%CI of RR. Lifestyle intervention (yes *vs* no), metformin intervention (yes *vs* no), and the product of both were included in the binary logistic regression model, and the product term was used to assess whether there was an interaction between lifestyle and metformin intervention.

Primary analyses were performed on an intention-to-treat basis. The primary clinical outcome was live birth. A 2 × 2 factorial design compared lifestyle intervention with no lifestyle intervention (groups A + C *vs* B + D) and metformin intervention with no metformin intervention (groups B + C *vs* A + D). Statistical analyses were performed using the Statistical Package for Social Sciences (SPSS V24.0, IBM Corp., Armonk, NY, USA), and a 2-sided *P*-value < 0.05 was used to establish statistical significance.

## Results

### Feasibility

**Fig. 1:**
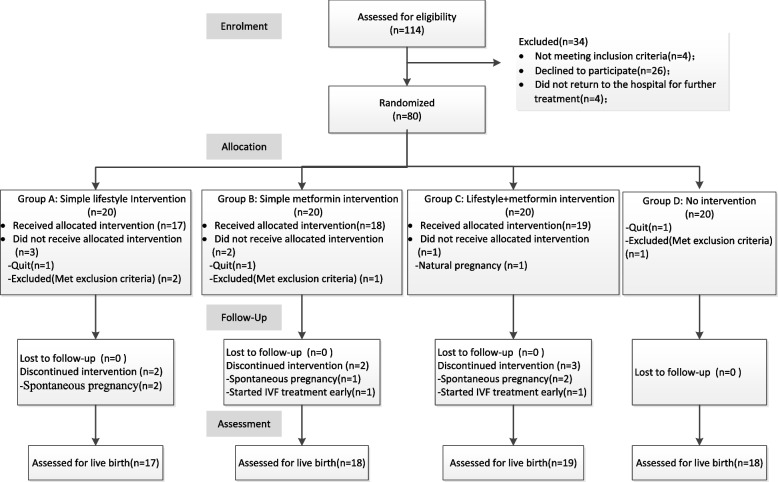
Consort flow diagram

Between June 2019 and April 2020, 1297 infertile women with BMI ≥ 25 kg/m^2^ were screened for eligibility, 114 (8.79%, 95% CI 7.25%–10.33%) were found eligible. Among eligible patients, 80 were randomised. The recruitment rate was 70.18% (95% CI:61.65%–78.70%). An average of 10 subjects were randomised each month. A total of 72 women received the allocated treatment. Of the remaining eight women, three withdrew informed consent, four met the exclusion criteria, and one had a spontaneous pregnancy before receiving the intervention. Of the 72 women, 71 received IVF/ICSI infertility treatment and one discontinued treatment due to a lack of financial resources. Of those who received IVF/ICSI, 100% had completed the follow-up of their first fresh embryo cycle (Fig. 1).

None of the participants crossed over from one group to another. Good compliance with metformin intervention, which is based on the international mainstream standard for the evaluation of medication compliance for chronic diseases [28] was observed in 91.89% (34/37) of the participants. Good compliance with the lifestyle intervention evaluated by the dietitian was observed in 94.44% (34/36) of the participants.

Approximately 77.78% (56/72) of the women started IVF/ICSI infertility treatment after the goal of the intervention was achieved or when the 12-week intervention was completed. Two women wanted to complete infertility treatment as soon as possible and therefore started IVF/ICSI treatment before the intervention goal was achieved. 14 women could not come to the hospital for some reason and did not start IVF/ICSI treatment until more than 12 weeks later, among eight women were unable to visit the hospital due to COVID-19 restrictions. All randomised participants completed the follow-up.

### Baseline characteristics

The baseline characteristics, including age, BMI, duration of infertility, and anti-Müllerian hormone (AMH) levels in the four groups were reported in Table 1. These participants had a mean age of 29.99 ± 3.30 years, with an average BMI of 27.84 ± 2.02 kg/m^2^ and average HOMA value of 3.86 ± 0.88.

**Table 1:** Baseline characteristics of the different allocation groups

	Group A (*n* = 17)	Group B (*n* = 18)	Group C (*n* = 19)	Group D (*n* = 18)
Age — years	31(30–34)	29(26.75–33)	31(27–33)	29(27–31.25)
Weight — kg	69.80(63.80–71.30)	65.75(64.28–68.75)	67.20(64.40–73.10)	68.65(64.38–73.10)
BMI — kg/m2	27.27(26.68–30.30)	26.66(25.98–28.00)	27.38(26.16–29.30)	28.35(26.42–29.27)
Waist circumference — cm	93.00(88.50–97.00)	92.00(86.25–96.25)	91.50(87.50–97.50)	95.00(90.00–97.00)
Hip circumference — cm	103.00(98.50–106.50)	100.00(98.00–103.00)	102.00(100.00–104.25)	103.00(98.00–107.00)
Duration of Infertility — years	3(2–6)	3(2–5)	3(2–5)	3(2–4)
AMH— ng/ml	1.84(1.35–4.09)	2.87(1.63–5.15)	3.42(2.1–4.84)	4.25(2.58–6.25)
AFC—n	16(12.50–38.50)	19(11–30)	25(19–30)	27.5(18.25–30.50)
HOMA value before intervention	3.67(3.05–4.74)	3.57(3.16–4.94)	3.85(3.40–4.10)	3.48(3.06–4.23)

For continuous variables median (interquartile ranges, IQR) is presented

*BMI* Body Mass Index, *AMH* Anti-Mullerian Hormone, *AFC* Antral Follicle Counting, *HOMA* Homeostasis Model Assessment

Group A: simple lifestyle intervention, Group B: simple metformin intervention, Group C: lifestyle + metformin intervention, Group D: no intervention

### Intervention outcomes

The results of the intervention based on the intention-to-treat analysis of the four groups were shown in Table 2. The efficacies of the lifestyle and metformin interventions were shown in Table 3. The numbers of participants who achieved the intervention goals in each group were shown in Supplemental Table 1.

A total of 36 women received lifestyle interventions (groups A and C), and 36 women did not receive lifestyle interventions (groups B and D). The weight loss percentages of those who received lifestyle intervention and those who did not were 7.07% (4.62%–9.17%) and 1.36% (0.36%–3.35%), respectively, with an MD (95%CI) of 5.29% (3.63%-6.95%). The rates of weight loss of 5–10% in the two groups were 72.22% and 8.33%, respectively, with an RR (95%CI) of 8.67 (2.88–26.09).

A total of 37 women received metformin intervention (groups B and C) and 35 women did not receive metformin intervention (groups A and D). The rates of HOMA value < 2.69 in those who received metformin intervention and those who did not were 67.57% and 35.29%, respectively, with an RR (95%CI) of 1.91 (1.15–3.18).

**Table 2:** Intervention outcomes

	Group A (*n* = 17)	Group B (*n* = 18)	Group C (*n* = 19)	Group D (*n* = 18)
Participants of loss weight 5–10%—n(%)	12(70.59)	2(11.11)	14(73.68)	1(5.56)
Participants of HOMA value below 2.69 after intervention— n(%)	7(41.18)	14(77.78)	11(57.89)	5(27.78)
Weight loss percentage — %	5.26(4.39–9.59)	2.40(0.72–3.9)	7.37(4.50–9.23)	0.71(0.10–1.58)
Reduced waist circumference — cm	7.00(1.00–9.50)	2.00(-1.25–3.63)	3.50(0.50–6.25)	0.75(-1.50–7.75)
Reduced hip circumference— cm	2.00(0.50–7.50)	1.00(-0.75–3.50)	1.50(-2.25–6.50)	1.00(0.00–1.75)

For categorical variables n (%) is presented. For continuous variables median (interquartile ranges, IQR) is presented

Group A: simple lifestyle intervention, Group B: simple metformin intervention, Group C: lifestyle + metformin intervention, Group D: no intervention

**Table 3:** Efficacy outcomes

	Lifestyle intervention		RR/ MD (95%CI)	Metformin intervention		RR/ MD (95%CI)
	YES (Groups A + C)	NO (Groups B + D)		YES (Groups B + C)	NO (Groups A + D)	
Participants of loss weight 5–10% — n(%)	26 (72.22)	3 (8.33)	8.67 (2.88–26.09)	16 (43.24	13 (37.14)	1.16 (0.66–2.05)
Participants of HOMA value below 2.69 after intervention—n(%)	18 (51.43)^a^	19 (52.77)	0.97 (0.62,1.52)	25(67.57)	12 (35.29)^a^	1.91 (1.15–3.18)
Weight loss percentage—%	7.07 (4.62–9.17)	1.36 (0.36–3.35)	5.29 (3.63–6.95)	4.50 (1.81–7.59)	1.60 (0.34–5.26)	1.43 (-0.60–3.48)
Reduced waist circumference—cm	5.00 (1.00–8.00)	1.50 (-1.25–7.00)	3.03 (-0.44–6.50)	2.00 (-0.75–4.75)	4.00 (0.00–8.00)	-1.67 (-4.95–1.61)
Reduced hip circumference —cm	2.00 (0.00–7.00)	1.00(0.00–2.50)	1.51 (-1.23–4.26)	1.00 (-1.00–4.00)	1.00 (0.00–2.50)	-0.20 (-2.72–2.32)

For categorical variables n (%) is presented. For continuous variables median (interquartile ranges, IQR) is presented

Group A: simple lifestyle intervention, Group B: simple metformin intervention, Group C: lifestyle + metformin intervention, Group D: no intervention

*HOMA* Homeostasis Model Assessment, *RR* Relative Risk, *MD* Mean difference

^a^Homa value of 1 subject was missing

### Clinical outcomes

The outcomes of controlled ovarian hyperstimulation and pregnancy in the four groups were shown in Table 4. The effects of lifestyle and metformin interventions were shown in Table 5. Compared to group D, RRs (95%CI) of groups A, B and C, were 0.64 (0.18–2.26), 1.40 (0.55–3.60) and 1.71 (0.71–4.12), respectively.

**Table 4:** Outcomes of controlled ovarian hyperstimulation and pregnancy

	Group A (*n* = 17)	Group B (*n* = 18)	Group C (*n* = 19)	Group D (*n* = 18)
Gonadotropin dose — IU	3225.00(2362.50–3984.38)	4381.25(3159.38–5334.38)	3675.00(2512.5–4087.5)	3150.00(2502.75–3993.75)
Endometrial thickness — mm	13.55(11.68–14.38)	13.70(13.15–14.55)	12.90(12.20–15.05)	13.55(11.95–14.88)
Spontaneous pregnancy — n	2	1	2	0
Oocytes retrieved — n	11.5(8.50–17.75)	10(8.00–18.75)	13(8.50–17.00)	14.5(9.00–19.50)
Embryos on day 3 — n	8(4–11)	6(4–13.50)	7(3–11.75)	8.5(5.75–12)
Cycles of embryos transferred— n	7	11	10	13
Cycles cancelled — n	8	6	7	5
Clinical pregnancy — n (including spontaneous pregnancy)	5	7^a^	9	8
Ongoing pregnancy — n (including spontaneous pregnancy)	3	7	9	6
Live birth — n (including spontaneous pregnancy)	3	7	9	5

For continuous variables median (interquartile ranges, IQR) is presented

Group A: simple lifestyle intervention, Group B: simple metformin intervention, Group C: lifestyle + metformin intervention, Group D: no intervention

^a^1case ectopic pregnancy wasn't included

**Table 5:** Effects of lifestyle intervention and metformin intervention (RR)

Lifestyle intervention	Metformin intervention	LBR	RR
No	No	27.78%	1
Yes	No	17.65%	0.64(0.18–2.26)
No	Yes	38.89%	1.40(0.55–3.60)
Yes	Yes	47.37%	1.71(0.71–4.12)
Lifestyle intervention (with vs without: 33.33%^a^ vs 33.33%^b^) RR: 1.00(0.52–1.92)			
Metformin intervention (with vs without: 43.24%^c^ vs 22.86%^d^) RR: 1.89(0.93–3.86)			
Interaction *P*-value for lifestyle × metformin:0.380			

Group A: simple lifestyle intervention, Group B: simple metformin intervention, Group C: lifestyle + metformin intervention, Group D: no intervention

^a^ LBR of groups A + C, 33.33% = (3 + 9)/(17 + 19)*100%

^b^ LBR of groups B + D, 36.11% = (7 + 5)/ (18 + 18) *100%

^c^ LBR of groups B + C, 43.24% = (7 + 9)/ (18 + 19) *100%

^d^ LBR of groups A + D, 25.71% = (3 + 5)/(17 + 18) *100%

The LBRs for groups A + C *vs* B + D were 33.33% (12/36) *vs* 33.33 (12/36) (RR = 1.00, 95%CI: 0.52–1.92). The LBRs for groups B + C *vs* A + D were 43.24% (16/37) *vs* 22.86% (8/35) (RR = 1.89, 95% CI:0.93–3.86). There was no evidence of an interaction between lifestyle factors and metformin use (*p* = 0.380). The pilot study was not sufficiently powered to detect differences in clinical outcomes.

### Complications and adverse events

Seven severe adverse events (SAE) requiring hospitalisation were reported, including one case of OHSS (in the lifestyle intervention group), one case of ectopic pregnancy (in the metformin intervention group), and five cases of miscarriage (two in the lifestyle intervention group and three in the non-intervention group). Considering that these SAEs were routine occurrence events of assisted reproduction, and the frequency of each group was not significantly higher than that of conventional treatment, it was considered that these SAEs may not be related to the intervention.

There were 16 cases of adverse drug reactions (ADR), mainly manifested as nausea, vomiting, and diarrhoea, of which seven occurred in the simple metformin intervention group and nine in the lifestyle + metformin intervention group. All 16 AE occurred after the use of metformin, and the severity of the events changed with the dosage adjustment; therefore, they were considered to be related to the metformin intervention. (see Table 6).

**Table 6:** Adverse events in the study

AE	Number	Groups in which AE occurred
OHSS	1	lifestyle intervention group
Ectopic pregnancy	1	metformin intervention group
Miscarriage	5	2 cases in the lifestyle intervention group and 3 cases in the non- intervention group
Gastrointestinal problems (e.g. nausea, vomiting, diarrhoea)	16^a^	7 cases in the simple metformin intervention group and 9 cases in the lifestyle + metformin intervention group

^a^These decreased metformin dose due to adverse events

## Discussion

In this pilot study, we demonstrated the feasibility of conducting a factorial-design RCT to evaluate the effect of lifestyle or metformin intervention and the interaction effect of the two interventions in infertile women with overweight/obesity and IR. Five of the six feasibility criteria regarding recruitment rate, number of recruitments per month,intervention compliance, and follow-up compliance were achieved as expected. The pilot study showed that only 77.78% (56/72) of women started IVF/ICSI infertility treatment after achieving the goal of the intervention or finishing a 12-week intervention, which was lower than the expected value (at least 80%). A major reason was that some subjects (50.0%, 8/16) were affected by the Covid-19 pandemic, which led to widespread lockdowns and prevented patients from returning to the hospital for planned treatment. Another main reason was that some subjects (31.3%, 5/16) had not yet reached the time to start IVF/ICSI infertility treatment (that was downregulation in the midluteal phase of the menstrual cycle) after achieving the goal of the intervention or finishing the 12-week intervention; therefore, they could not start IVF/ICSI treatment immediately after ending the intervention. In addition, some patients (18.8%, 3/16) voluntarily requested early or delayed IVF/ICSI treatment. Excluding the impact of the pandemic, this reminds us that initiating lifestyle (and/or) metformin intervention in conjunction with the subject’s menstrual cycle and keeping subjects fully informed may increase the number of patients who start IVF/ICSI treatment as planned, thereby improving intervention compliance.

The pilot study showed the eligibility rate was low, at 8.79% (95% CI 7.25%–10.33%). A total of 1297 infertile women with BMI ≥ 25 kg/m^2^ were screened for eligibility, 114 were found eligible. The main reasons for the low eligibility rate were as follows: first, many overweight/obese patients had IR or PCOS, accounting for approximately 44.6% (578/1296), who did not meet the inclusion criteria; second, a high proportion of patients (22.8%, 296/1296) did not return to the hospital for further examination and treatment; and third, the study set many exclusion criteria. Patients with adenomyosis, uterine adhesions, or other uterine factors (11.2%, 145/1296) were excluded from the study. Strict inclusion can create a good balance between groups, but reduces the eligibility rate and limits the extension of research results. Whether to delete some exclusion criteria, such as endometriosis or uterine abnormalities, should be considered by the researchers.

Among the 80 randomised subjects in the pilot study, three withdrew informed consent and four met the exclusion criteria. This may be due to the investigator’s unfamiliarity with the protocol and participants not being fully informed. To reduce randomisation in error, these personnel who perform screening and informed consent interviews need to be retrained. Healthcare personnel should carefully verify the inclusion criteria, ensure that subjects are well informed, and give them sufficient time to consider participation.

In addition, we found that some factors, such as AMH and AFC, have been unbalanced between groups after randomisation, which may have been caused by data fluctuations in the small-sample pilot study. It is undeniable that some characteristics may remain unbalanced between groups after randomisation in a formal study. To maintain a good balance between groups, we will use a stratified randomised method to balance some important characteristics (such as overweight vs. obesity) in the formal trial; however, if there are still differences between groups in some characteristics, we will use multivariable analysis to adjust for these factors.

This study showed that lifestyle interventions preceding infertility treatment resulted in substantial weight loss and slight improvement in IR, whereas metformin intervention improved insulin sensitivity and had a modest impact on weight loss. It was worth noting that this study also found that lifestyle combined with metformin intervention reduced BMI and insulin levels to a greater degree than a single intervention, which was consistent with the findings of Pasquali et al. [29–31]. some studies [17] have reported that the addition of metformin to patients on a diet or lifestyle program did not contribute to further weight loss. However, these conclusions should be interpreted with caution, as Nieuwenhuis-Ruifrok advocated that adequately powered RCTs are required to confirm the findings and assess whether the addition of metformin therapy to a structured lifestyle modification program might contribute to greater weight loss and IR improvement. In terms of the effect of live birth, the combined intervention also had the highest LBR (RR = 1.71, 95% CI:0.71–4.12), which was consistent with the effects of weight loss and insulin regulation. However, RCTS with large sample size are required for further confirmation.

### Strengths and limitations

#### Strengths

The major strength of this study was the factorial design. Studying two intervention treatments simultaneously within one trial is more efficient than the traditional separate treatment evaluation, as both the sample size and duration of the trial will be smaller. The interaction effect of two interventions can also be studied using a factorial design.

In addition, many patients receive infertility treatment and delivery not in the same hospitals, and we followed up by telephone call to obtain live birth outcomes. To guarantee the reliability of the results, we asked participants to send their relevant medical records online. The pilot study showed that the follow-up method was feasible.

#### Limitations

The study was not blinded. To process data realistically and present real results, formal trials should consider assessor blinding. Another limitation was that the study was conducted in a single centre; if future definitive trials are conducted in multiple centres, the criteria for recruitment, randomisation, intervention, and follow-up still need to be unified.

## Conclusions

In conclusion, it is feasible to conduct a factorial RCT to evaluate the effects of lifestyle or metformin interventions before IVF/ICSI treatment in overweight/obese infertile women with IR. The pilot study found that lifestyle intervention could significantly reduce body weight and metformin intervention could regulate IR; however, neither intervention was found to improve LBR in infertility treatment. Owing to the small sample size, we could not draw conclusions regarding the effectiveness of the two intervention methods. Therefore, a RCT with a large sample size is necessary.

### Supplementary Information

**Additional file 1:Supplemental table 1.** Numbers of participants who achieved the goal of the intervention in each group.

## Data Availability

The datasets used and analyzed during the current study and other materials are available from the corresponding author on reasonable request.

## Abbreviations

RCT: Randomised Controlled Trial
PCOS: Polycystic Ovarian Syndrome
IR: Insulin Resistance
BMI: Body Mass Index
HOMA: Homeostasis Model Assessment
OGTT: Oral Glucose Tolerance Test
IRT: Insulin Release Test
IVF: In Vitro Fertilization
ICSI: Intracytoplasmic Sperm Injection
PGT: Preimplantation Genetic Tests
FSH: Follicle-Stimulating Hormone
AMH: Anti-Mullerian hormone
HCG: Human chorionic gonadotropin
HMG: Human Menopausal Gonadotropin
OPR: Ongoing Pregnancy Rate
LBR: Live Birth Rate
CI: Confidence Interval
ME: Margin of error
RR: Relative Risk
MD: Mean difference
SD: Standard Deviations
SAE: Severe Adverse Events
ADR: Adverse Drug Reaction
COVID-19: Coronavirus disease 2019
APP: Application
