# Supplementary material for: Effect of lifestyle or metformin interventions before IVF/ICSI treatment on infertile women with overweight/obese and insulin resistance: a factorial design randomised controlled pilot trial
Source: Pilot Feasibility Stud. 2023 Sep 12;9:160. doi: 10.1186/s40814-023-01388-x (PMC10496164; doi:10.1186/s40814-023-01388-x)
Supplement: Supplementary file 1 — Additional file 1:Supplemental table 1. Numbers of participants who achieved the goal of the intervention in each group. [file 40814_2023_1388_MOESM1_ESM.docx]

Supplemental table 1 Numbers of participants who achieved the goal of the intervention in each group

|  | Group A  （n=17） | Group B  （n=18） | Group C  （n=19） | Group D  （n=18） |
| --- | --- | --- | --- | --- |
| Participants achieved weight loss 5-10% only—n | 6 | 0 | 5 | 0 |
| Participants achieved HOMA value below 2.69 only—n | 1 | 12 | 2 | 4 |
| Participants achieved both weight loss 5-10% and HOMA value below 2.69—n | 6 | 2 | 9 | 1 |
